# Trauma-related postoperative redisplacement after tension-band fixation for patellar fracture in Parkinson's disease: a case report and focused literature review

**DOI:** 10.3389/fsurg.2026.1791697

**Published:** 2026-08-27

**Authors:** Shaowei Sun, Zhixiang Gao, Longyun Li, Lifu Wang, Cong Xiao

**Affiliations:** 1 North Sichuan Medical College, Nanchong, China; 2 Department of Orthopedics, The Third Hospital of Mianyang Sichuan Mental Health Center, Mianyang, China

**Keywords:** case report, Parkinson's disease, patellar fracture, postoperative redisplacement, revision surgery, tension-band fixation

## Abstract

**Rationale:**

Patellar fractures in patients with Parkinson's disease (PD) are rarely reported, and evidence regarding optimal fixation and postoperative management remains limited. In this population, recurrent falls, abnormal muscle tone, and impaired bone quality may increase the risk of treatment failure.

**Patient concerns:**

A 78-year-old man with long-standing PD presented with a patellar fracture after a low-energy fall and subsequently developed trauma-related postoperative redisplacement after a recurrent fall following primary tension-band fixation.

**Diagnoses:**

The patient was diagnosed with a displaced left patellar fracture (AO/OTA 34-C3) complicated by secondary traumatic redisplacement after a postoperative fall.

**Interventions:**

The patient initially underwent tension-band fixation using two patellar pins, supplemented by suture-based soft-tissue reinforcement. After postoperative traumatic redisplacement, revision surgery with augmented fixation and additional suture-based soft-tissue reinforcement was performed.

**Outcomes:**

Radiographic follow-up demonstrated partial fracture union with residual nonunion and fragment avulsion. After revision surgery, knee range of motion (ROM) was 0°–20° at postoperative days 1–3, and at 1 month the visual analogue scale pain score was 1, with Lysholm and Bostman scores of 22 and 5, respectively. At 9 months, ROM was 0° to 30°, with full extension to 0°. Although CT showed partial union with residual nonunion and fragment avulsion, the patient was able to ambulate independently with a slow gait and perform daily activities. No Lysholm, Bostman, or Knee Society Score was obtained at the final follow-up.

**Lessons:**

This case suggests that patellar fracture fixation in patients with PD may be particularly challenging. Recurrent falls and other PD-related factors may contribute to postoperative redisplacement and construct compromise. This case also highlights the need to consider careful surgical planning, appropriate postoperative protection, fall-risk mitigation, and bone health evaluation in similar high-risk patients.

## 1 Introduction

PD is a common neurodegenerative disorder in older adults, characterized by motor symptoms such as resting tremor, rigidity, bradykinesia, and postural instability. These symptoms are associated with impaired balance control and a higher risk of falls, significantly increasing the likelihood of fractures in this population (1). Additionally, patients with PD often experience sarcopenia and osteoporosis, leading to reduced bone mineral density (BMD) and increased skeletal fragility (2–4). Consequently, fractures are more common in patients with PD than in the general population and are associated with poorer outcomes (5, 6).

Patellar fractures are rare, accounting for approximately 1% of all fractures (7, 8). However, treating fractures in PD patients is particularly challenging due to the altered bone quality and the unique biomechanical issues associated with PD. Muscle rigidity and abnormal motor control can complicate fixation and rehabilitation. Literature on postoperative redisplacement or fixation-related complications after patellar fracture in patients with PD is limited (6).

This report describes a case in which a patellar fracture in a patient with PD was complicated by trauma-related postoperative redisplacement after a recurrent fall following traditional tension-band fixation. While tension-band fixation is commonly used for such fractures (9), its performance may be compromised in PD patients due to increased muscle tone and bone fragility (10). Following this trauma-related complication, revision surgery was performed using an augmented fixation strategy, including a crossed configuration of patellar pins and additional suture-based soft-tissue reinforcement involving both the patellar ligament and quadriceps tendon. These adjustments aimed to improve the stability of the fixation and better accommodate the challenges posed by the patient's condition.

## 2 Case presentation

A 78-year-old man with an approximately 20-year history of PD, classified as Hoehn-Yahr stage III, underwent preoperative neurological consultation before both surgeries. His anti-parkinsonian medication regimen included levodopa/benserazide 125 mg three times daily, amantadine 0.2 g three times daily, and trihexyphenidyl 2 mg three times daily. Over the preceding two years, he experienced progressively worsening balance instability. One day before admission, the patient fell from a standing position due to left lower limb weakness, without direct trauma to the left knee.

**Figure 1:**
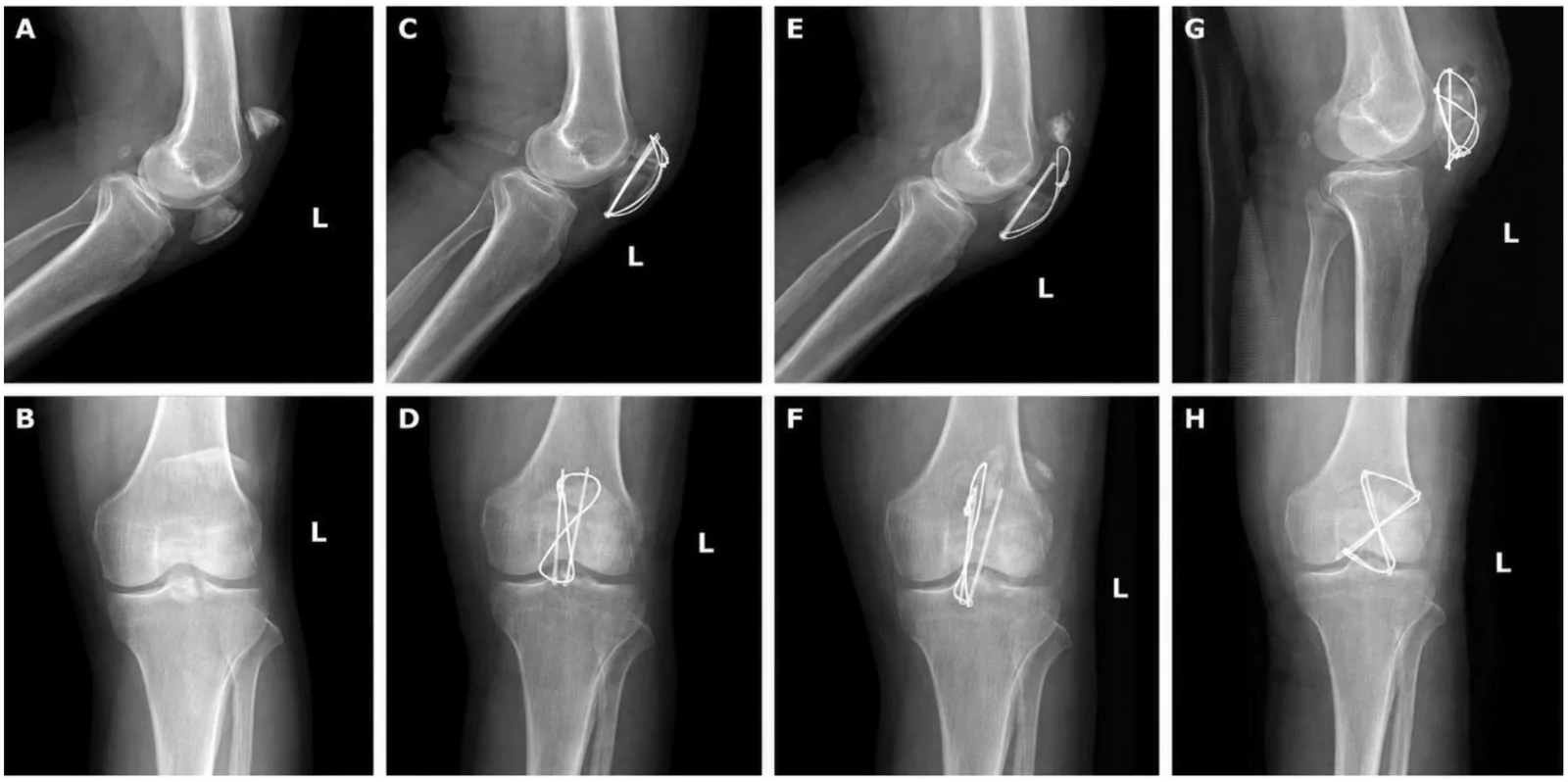
Radiographic evaluation during the clinical course. **(A,B)** Preoperative lateral and anteroposterior radiographs showing the initial left patellar fracture. **(C,D)** Immediate postoperative lateral and anteroposterior radiographs demonstrating satisfactory reduction and alignment after primary fixation. **(E,F)** Lateral and anteroposterior radiographs after the postoperative fall, showing secondary traumatic redisplacement with comminution involving the proximal pole of the patella. **(G,H)** Post-revision lateral and anteroposterior radiographs showing restored alignment and re-fixation stability.

Physical examination revealed swelling and deformity of the left knee, localized tenderness over the patella, and limited active extension. Anteroposterior and lateral radiographs confirmed a displaced left patellar fracture, classified as AO/OTA 34-C3, with comminution of the superior pole that was completely separated from the distal fragment. Displacement was approximately 2 cm in extension and 3 cm in flexion (Figures 1A,B). The extensor mechanism appeared intact, and intraoperative findings confirmed no defects in the patellar tendon, quadriceps tendon, or retinaculum. Although DEXA was not performed, the imaging report described osteoporosis on radiographic assessment, and CT findings were consistent with reduced bone quality. The patient had been receiving calcium and vitamin D3 supplementation, which was continued after surgery. No anti-osteoporotic pharmacotherapy was initiated during the observed treatment period. The absence of formal DEXA assessment and anti-osteoporotic pharmacotherapy was recognized as a limitation of the management in this case.

Following routine preoperative assessment and consultation, the patient underwent surgery under general anesthesia. In the index procedure, two parallel patellar pins were inserted from the inferior pole toward the superior pole, and a tension-band construct was applied. Additional reinforcement was provided by continuous locking Covidien Ti-Cron™ coated braided polyester sutures made of polyethylene terephthalate, USP size 2/metric size 5, 75 cm in length, through the patellar ligament. Postoperative anteroposterior and lateral radiographs showed satisfactory reduction and alignment after primary fixation (Figures 1C,D).

**Figure 2:**
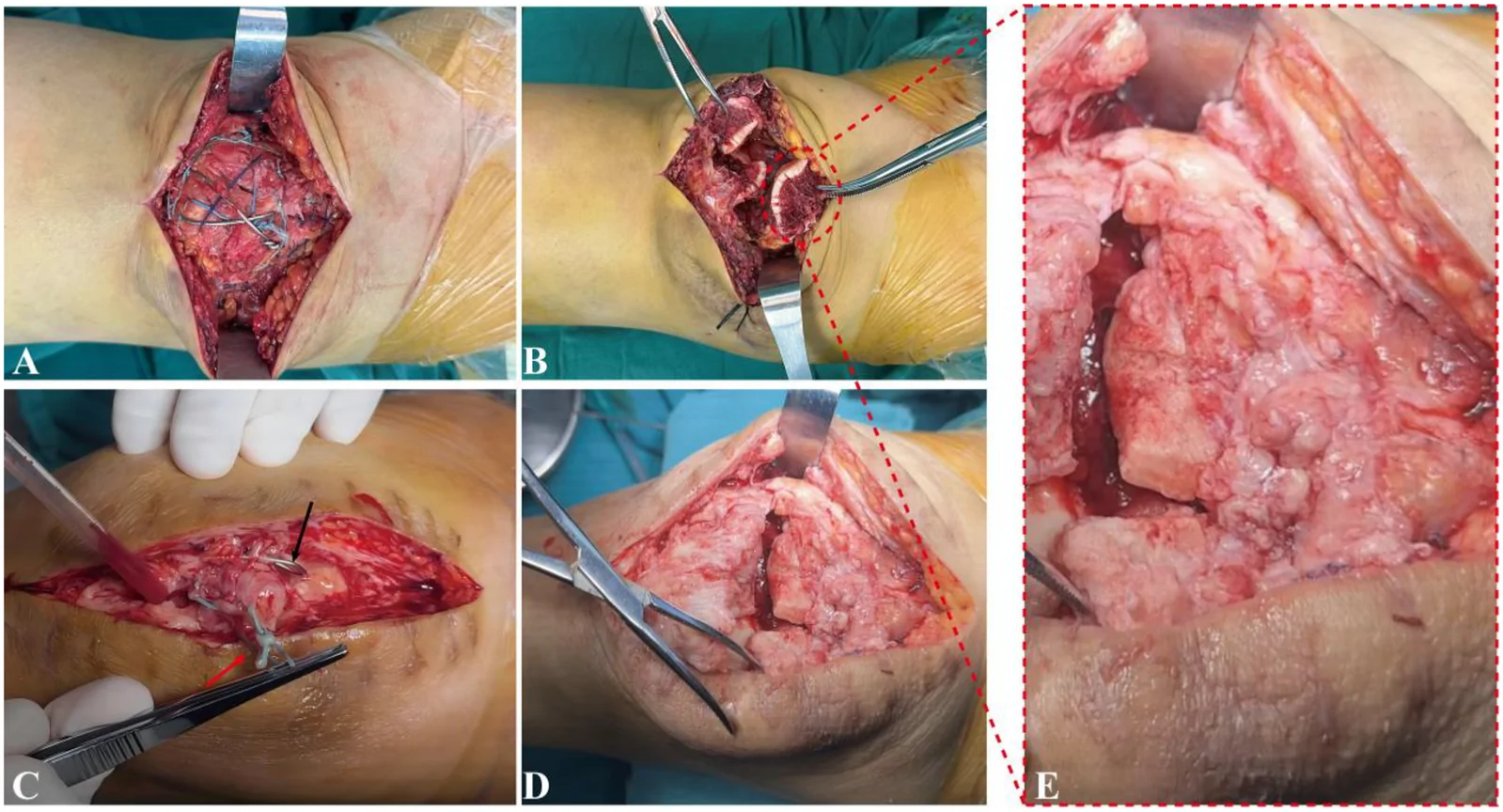
Representative intraoperative photographs during revision surgery. **(A)** Exposure during revision surgery after removal of the previous fixation, with preparation for augmented fixation and continuous locking suture reinforcement. **(B)** Intraoperative view demonstrating fragmentation of the superior pole into two fragments. **(C)** Intraoperative image showing wire/suture cut-through of the fracture fragments. **(D,E)** Intraoperative photographs further illustrating fracture-site refreshment, fragment preparation, and superior-pole comminution before final re-fixation.

Twenty days after the index surgery, on July 25, 2024, the patient sustained another fall, resulting in redisplacement of the fracture fragments (Figures 1E,F). Revision surgery was performed, with a preoperative neurological consultation. Intraoperative findings included wire and suture cut-through of the fracture fragments (Figure 2C), as well as further fragmentation of the superior pole into two separate fragments (Figures 2B,D,E). During the revision, two crossed patellar pins were inserted from the superior pole toward the inferior pole, and a tension-band construct was applied. Additional reinforcement was achieved by passing locking Covidien Ti-Cron™ coated braided polyester sutures made of polyethylene terephthalate, USP size 2/metric size 5, 75 cm in length, through both the patellar ligament and quadriceps tendon to augment the fixation. Representative intraoperative photographs showing the key intraoperative findings during revision surgery, including removal of the previous fixation, wire/suture cut-through, fracture-site refreshment, and superior-pole fragmentation, are shown in Figure 2. Complete photographs of the final re-fixation step were not available because of the retrospective nature of this case; therefore, the re-fixation technique is described in detail in the text. Post-revision radiographs showed restored alignment and construct stability (Figures 1G,H). The same fixation materials, including two 2.0 × 70 mm patellar pins, a 1.2 mm tension-band wire, and Covidien Ti-Cron™ coated braided polyester sutures were used in both procedures.

Following both surgeries, weight-bearing was restricted, and the knee was immobilized in a plaster cast at approximately 20°–30° of flexion. Postoperative day 1 included ankle-pump and toe-motion exercises. At 1 month postoperatively, the visual analogue scale pain score was 1, and the Lysholm and Bostman scores were 22 and 5, respectively. Knee mobilization was initiated under the guidance of a rehabilitation physician at 6 weeks postoperatively, and assisted out-of-bed ambulation was permitted at 3 months. The anti-parkinsonian regimen was adjusted to include levodopa/benserazide 125 mg three times daily, plus 62.5 mg at bedtime, trihexyphenidyl 2 mg three times daily, amantadine 0.1 g three times daily, pramipexole 0.125 mg three times daily, and baclofen 5 mg three times daily to reduce spasm and rigidity.

**Figure 3:**
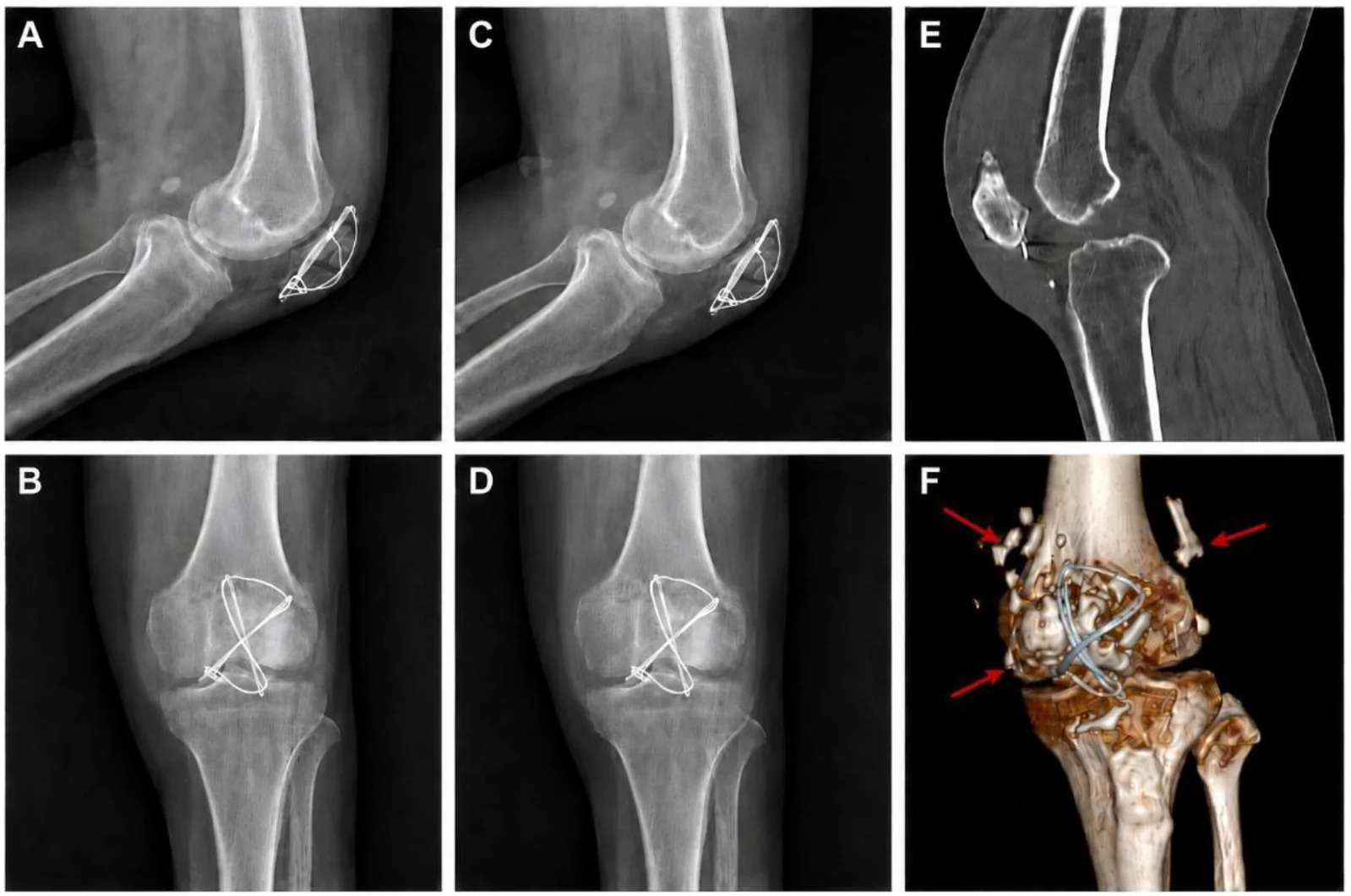
Postoperative radiographic and CT follow-up after revision surgery. **(A,B)** Lateral and anteroposterior radiographs at 1 month after revision surgery, showing maintained fixation without obvious redisplacement. **(C,D)** Lateral and anteroposterior radiographs at 2 months after revision surgery, showing no obvious progression of fragment displacement. **(E,F)** CT images at the final follow-up, demonstrating partial fracture union with residual nonunion and displaced/avulsed bone fragments around the patella.

Follow-up radiographs at one and two months after revision showed maintained fixation with no recurrent displacement (Figures 3A–D). At 9 months postoperatively, CT revealed partial fracture union with residual nonunion, an avulsed fragment, and small isolated bone fragments near the patella, likely resulting from the previous wire cut-through (Figures 3E,F). Clinically, the knee ROM was 0°–30°, with apparent full extension to 0°; however, extensor lag was not quantitatively measured at final follow-up, so the finding of full extension should be interpreted cautiously. Despite incomplete radiographic healing, the patient could ambulate independently with a slow gait and perform daily activities. Given the minimal functional impairment and limited expected benefit from further surgery, no additional intervention was performed. The clinical timeline is presented in Supplementary Figure 1.

## 3 Discussion

### 3.1 Patellar fractures and PD

Patients with PD have an increased risk of fractures and may experience less favorable outcomes after fracture (11, 12). However, reports specifically addressing patellar fracture fixation in this population remain extremely limited. The present case highlights trauma-related postoperative redisplacement after standard tension-band fixation following a patellar fracture in a PD patient, which underscores the potential vulnerability of conventional fixation constructs in this high-risk setting.

### 3.2 Contributors to postoperative redisplacement and construct compromise

In this case, the immediate cause of fixation failure was a recurrent fall occurring 20 days after the index procedure. PD patients are particularly prone to recurrent falls due to postural instability and impaired motor control (13–15). These falls can subject fixation constructs to repeated mechanical stress, increasing the risk of early failure. In addition to fall-related trauma, several other patient-related factors may have contributed. For instance, abnormal muscle tone, including persistent quadriceps hypertonicity, could increase tensile loading across the patella (16, 17). Impaired bone quality may also reduce resistance to wire or suture cut-through. Although formal DEXA-based BMD assessment was not available, the imaging report clearly described osteoporosis, suggesting that osteoporosis may have contributed to wire or suture cut-through. These elements should be considered as plausible contributors rather than definitive mechanisms.

### 3.3 Literature review and focused overview

Patellar fractures in patients with PD or parkinsonism are rarely reported. To provide a reproducible focused literature overview, we performed a PubMed search on August 9, 2026, from database inception to August 9, 2026, using the following search strategy: (“Parkinson disease” OR “Parkinson's disease” OR Parkinson* OR parkinsonism) AND (patella OR patellar) AND (fracture OR fractures). No language restriction was applied. We included case reports, case series, or clinical studies describing patellar fractures in patients with PD or parkinsonism, including spontaneous or low-energy patellar fractures and post-TKA patellar fractures. Studies involving fractures at anatomical sites other than the patella, cases without PD or parkinsonism, non-clinical studies, and reviews without original cases were excluded. The reference lists of the included articles were also screened manually.

This focused search identified two published reports of patellar fractures in patients with PD or parkinsonism: one non-TKA case of spontaneous bilateral patellar fracture in an elderly PD patient and one post-TKA patellar fracture report (17, 18). The included reports are summarized in Table 1. Due to the scarcity of cases specifically addressing patellar fractures in PD, this review should be considered a focused clinical overview rather than a systematic review.

**Table 1:** Published reports of patellar fractures in patients with PD/parkinsonism.

Author/year	PD/parkinsonism context	Fracture/treatment	Outcome
Moretti et al., 2008 (18)	85-year-old woman with PD, osteoporosis, and diffuse degenerative osteoarthritis	Bilateral patellar fractures after repeated falls without direct knee trauma; conservative treatment	Good active and passive knee ROM (95°/0°/0°); resumed daily living activities and autonomous ambulation
Seijas et al., 2009 (17)	75-year-old man with early PD after TKA without patellar resurfacing	Displaced patellar fracture detected 2 months after surgery; conservative treatment	Knee ROM maintained at -15° to 100°; pain-free and walking with a cane at 4-year follow-up

Taken together, the limited available literature suggests that patellar fractures in patients with PD may occur in the context of recurrent falls, impaired bone quality, abnormal muscle tone, and altered biomechanics (10, 12, 15, 18). These factors may increase both fracture susceptibility and the risk of fixation-related complications, particularly in elderly patients or those undergoing lower-extremity procedures such as TKA (17, 19–21). Therefore, individualized fixation strategies and cautious rehabilitation protocols should be considered in this high-risk population (22–24).

### 3.4 Surgical strategy and biomechanical considerations

Tension-band fixation is widely used for patellar fractures, but its effectiveness may be influenced by fracture pattern, fixation configuration, and technical factors related to modified tension-band wiring (9, 25–28). In patients with impaired bone quality and neuromuscular disorders such as PD, fixation stability may be further challenged by osteoporosis, abnormal muscle tone, and recurrent trauma (10, 26). The trauma-related redisplacement observed after the index procedure raised important concerns regarding fixation stability in the setting of comminution, impaired bone quality, and PD-related abnormal muscle tone.

In the revision procedure, the fixation strategy was modified in two main ways. First, two patellar pins were inserted in a crossed configuration with the intention of engaging multiple fragments and providing better control of rotational and multidirectional displacement (27). Second, the soft-tissue reinforcement was expanded from the patellar ligament alone to both the patellar ligament and quadriceps tendon.

We revised our interpretation of the tension-band repositioning. Although the tension-band construct was placed closer to the superior pole during revision to adapt to the residual fragment configuration, the superior pole had already become comminuted and therefore could not be regarded as a uniformly reliable anchoring zone. In this context, the improved stability after revision should not be attributed solely to the more proximal wire position. Rather, the maintenance of fracture alignment during the early postoperative period after revision was more likely related to the combined effect of crossed-pin fixation, more comprehensive soft-tissue reinforcement involving the quadriceps tendon and patellar ligament, and strict postoperative immobilization and protected rehabilitation.

This case therefore suggests that, in PD patients with comminuted patellar fractures and poor bone quality, fixation strategies should avoid relying exclusively on wire purchase in small or osteoporotic fragments. Additional fragment control and soft-tissue augmentation may be particularly important when the extensor mechanism is exposed to persistent abnormal tensile forces generated by rigidity or spasm.

In the index surgery, reinforcement was limited to the patellar ligament, where Covidien Ti-Cron™ coated braided polyester sutures were applied to provide additional stability. However, this reinforcement did not prevent subsequent redisplacement after the recurrent fall. In the index procedure, reinforcement was limited to the patellar ligament and did not incorporate the quadriceps tendon or circumferential soft-tissue augmentation.

In contrast, the revision procedure incorporated both the patellar ligament and quadriceps tendon into the reinforcement construct. In addition to reinforcing the patellar ligament, the quadriceps tendon was incorporated into the reinforcement construct. Covidien Ti-Cron™ coated braided polyester sutures were passed through both the patellar ligament and quadriceps tendon and then wrapped around the patella to create a more robust fixation. This dual-layer reinforcement may have provided additional resistance against the abnormal tensile forces generated by the quadriceps, thereby contributing to improved construct stability.

### 3.5 Perioperative management

Beyond the choice of fixation technique, comprehensive perioperative management is crucial. In this case, neurological consultation and adjustments to anti-parkinsonian medication were made to help reduce rigidity and improve motor control. Fall-risk mitigation strategies, such as assisted mobilization and environmental precautions, are particularly important in the early postoperative period. Bone health management is also an important lesson from this case. Although the imaging report suggested osteoporosis and the patient continued calcium and vitamin D3 supplementation, formal DEXA assessment and anti-osteoporotic pharmacotherapy were not performed during the observed treatment course. Therefore, this case should be interpreted not as evidence that bone health management was adequately implemented, but as a reminder that early osteoporosis assessment and individualized anti-osteoporotic treatment should be considered in similar high-risk patients, where appropriate (3). Rehabilitation protocols must balance early mobilization with protection of the fixation construct, as excessive mechanical loading could lead to redisplacement or construct compromise in this patient population (9, 24).

### 3.6 Radiographic findings and descriptive functional status

At the final follow-up on April 6, 2025, approximately 9 months after the initial injury and approximately 8 months after revision surgery, CT demonstrated partial fracture union with residual nonunion and avulsed fragments. Clinically, the patient was able to ambulate independently with a slow gait and perform activities of daily living, despite limited knee flexion. Knee ROM was 0°–30° at the final follow-up; however, extensor lag was not quantitatively measured, so the statement of full extension to 0° should be interpreted cautiously.

Importantly, standardized functional scores, such as the Lysholm score, Bostman score, or Knee Society Score, were not obtained at the final follow-up. Therefore, the apparent discordance between radiographic findings and functional status should be interpreted only as a descriptive clinical observation rather than as an objectively quantified functional outcome.

In this elderly patient with long-standing PD, no further surgery was performed because the patient had minimal subjective functional impairment, maintained independent ambulation, and was considered unlikely to gain substantial additional benefit from another procedure. Management of patellar delayed union or nonunion should be individualized according to symptoms, extensor mechanism function, functional demands, and the overall condition of the patient (29, 30). Thus, the decision in this case reflected individualized, patient-centered management rather than evidence that incomplete radiographic union generally leads to acceptable function.

### 3.7 Limitations of the study

This report has several limitations. First, it describes a single case, and the findings cannot be generalized to all patients with PD or to all patellar fractures in this population. Second, formal DEXA-based BMD assessment and objective neuromuscular measurements were not available, limiting the ability to draw definitive conclusions about the mechanisms behind postoperative redisplacement and construct compromise. Although the imaging report described osteoporosis, the absence of DEXA assessment and anti-osteoporotic pharmacotherapy should be recognized as a limitation of the management in this case. Third, functional outcomes were not fully assessed with standardized patient-reported or clinician-reported outcome measures. In particular, although the patient was able to ambulate independently at 9 months, no final Lysholm, Bostman, or Knee Society Score was obtained; therefore, the functional status at final follow-up could only be described clinically and was not objectively quantified. Fourth, extensor lag was not quantitatively measured at final follow-up, so the statement of full extension to 0° should be interpreted cautiously. Lastly, the literature review was focused rather than systematic and was constrained by the limited and heterogeneous nature of available studies on this subject.

## 4 Conclusion

This case highlights the complexity of managing patellar fractures in patients with PD, emphasizing the potential pitfalls of traditional fixation techniques when recurrent trauma, impaired bone quality, and abnormal muscle tone coexist, as well as the value of biomechanical considerations in surgical planning. The revised surgical approach requires further evaluation in larger clinical studies before its broader applicability and effectiveness can be determined.

## Data Availability

The original contributions presented in the study are included in the article/Supplementary Material, further inquiries can be directed to the corresponding author.

## Supplementary material

Supplementary Figure 1 The timeline for this patient.
